# Epilepsy Detection Based on Variational Mode Decomposition and Improved Sample Entropy

**DOI:** 10.1155/2022/6180441

**Published:** 2022-01-18

**Authors:** Yandong Ru, Jinbao Li, Hangyu Chen, Jiacheng Li

**Affiliations:** ^1^College of Electronic Engineering, Heilongjiang University, Harbin 150006, China; ^2^College of Electronic and Information Engineering, Heilongjiang University of Science and Technology, Harbin 150027, China; ^3^Shandong Artificial Intelligence Institute, Qilu University of Technology (Shandong Academy of Science), Jinan 250013, China

## Abstract

Epilepsy detection based on electroencephalogram (EEG) signal is of great significance to diagnosis and treatment of epilepsy. The denoised EEG signal is adopted by most traditional epilepsy detection methods. But due to nonideal denoising ability, the loss of local information and residual noise will occur, resulting in detection performance degradation. To solve the problem, the paper proposed an epilepsy detection method in noisy environment. Although epileptic signals and nonepileptic signals have some discrimination, they need to overcome the interference of noise. Hence, the improved sample entropy and phase synchronization indexes of corresponding 2 intrinsic mode functions (IMFs) caused by variational mode decomposition (VMD) are proposed as features, which can reduce the impact of noise on detection performance. The experimental results show that the accuracy, sensitivity, and specificity are 91.78%, 91.27%, and 93.61%, respectively. It can be used as an auxiliary method for clinical treatment of epilepsy.

## 1. Introduction

Epilepsy is one of the common nervous system diseases affecting about 60 million people around the world [1]. Epilepsy detection results are one of the main basis for neurosurgeons to treat epilepsy. Traditional epilepsy detection is completed by neurosurgeons according to their own clinical experience by observing the electroencephalogram (EEG) [2]. This method not only takes a lot of time but also depends on the subjective judgment of neurosurgeons. Therefore, the realization of automatic high performance epilepsy detection is the main research direction for scholars [3].

As far as the authors know, the denoised EEG signals are widely used to detect epilepsy. But the epilepsy detection method based on denoised signal is limited in practical application because of the nonideal denoising. The denoising methods are divided into two classes. One is completed by bandpass filter based on the assumption that signal and noise live in different frequency bands. But boundary effect of filter causes poor filtering effect near the cut-off frequency. The other is to identify noise based on the assumption that noise and signal come from different sources. The independent component analysis method (ICA) is an outstanding representative of this class [4]. ICA officially states that it can filter out 95% of the noise, but the filtering effect of this method will become worse with the reduction of channels. In addition, ICA will consume a lot of time and cannot achieve real-time epilepsy detection. At the same time, the 2 classes of denoising methods will filter out some epileptic signals by mistake sometimes, resulting in the loss of epileptic information. To avoid the phenomenon, the paper realized epilepsy detection based on the complete signals in noisy environment.

EEG is a complex physiological phenomenon produced by the interaction of different tissues and organs. The nonlinear method can accurately describe the physiological features and obtain more information close to the real state of brain regulation [5, 6]. The most common nonlinear analysis methods include correlation dimension, Lyapunov exponent, and sample entropy. Because correlation dimension and Lyapunov exponent have certain requirements on data length [7], sample entropy is widely used for EEG analysis at present [8]. As far as authors know, sample entropy is obtained based on denoised signal in epilepsy detection so far. The sample entropy may be different from the real sample entropy due to the nonideal denoising. At the same time, the sample entropy represents the overall complexity of signal but lacks local information. The paper utilizes changing trend of local sample entropy to improve sample entropy, so that the improved sample entropy can represent overall complexity and local complexity, which can truly represent the characters of signals in noisy environment.

Phase synchronization is the result of brain nerve interaction, which can represent small dynamic changes because of its high sensitivity [9, 10]. In clinical application, neurosurgeons will determine the type of epilepsy according to the features in different frequency bands so as to make a treatment plan [11]. The frequency bands will be different because of the individual difference of patients. Sometimes, the bandpass filter can not eliminate the influence. Variational mode decomposition (VMD) solves the problem of frequency band decomposition as an adaptive decomposition method [12]. In the paper, phase synchronization index is selected as measurement method of phase synchronization. The signals from channel FZCZ and channel CZPZ are decomposed into 6 IMFs living in different frequency bands by VMD, respectively. 5 phase synchronization indexes of corresponding 2 IMFs (excluding IMF6) from different channels are selected for epilepsy detection because of their remarkable ability of epilepsy detection in noisy environment.

In the paper, the improved sample entropy and phase synchronization indexes are adopted in noisy environment. The main contributions can be summarized as follows.

- The improved sample entropy is proposed in noisy environment. It overcomes the shortcoming that the sample entropy cannot represent the real complexity of partial signals because of the local special signals sometimes. Comparing to traditional sample entropy, the improved sample entropy has stronger epilepsy detection ability in noisy environment.
- In order to obtain features that can be used by neurologists, frequency band decomposition is realized by VMD. It can solve the problem that fixed frequency band cannot be extracted due to individual differences of patients. The 5 phase synchronization indexes between corresponding 2 IMFs (excluding IMF6) are proved to have strong epilepsy detection ability in noisy environment.
- As far as the authors know, this paper realized epilepsy detection in noisy environment for the first time. It avoids epilepsy information loss in the process of filtering.

The remaining of the paper is organized as follows. Section 2 reviews the block diagram of epilepsy detection and the principle of adopted method in the paper.  Section 3 reviews the experimental processing and results, which contains feature extraction, feature analysis, and realization of epilepsy detection.  Section 4 reviews a brief conclusion and research direction in the future.

## 2. Principle and Methods

### 2.1. Overall Structure of Epilepsy Detection

**Figure 1:**
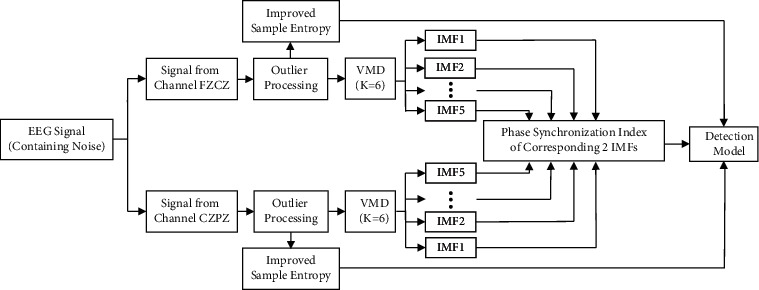
Block diagram of epilepsy detection.

In the paper, the signals from channel FZCZ and channel CZPZ are selected and processed by outlier processing. The improved sample entropy is obtained which can truly represent the complexity of the signals in noisy environment. The signals from the 2 channels are divided into 6 IMFs by VMD, respectively. 5 phase synchronization indexes of corresponding 2 IMFs (excluding IMF6) are selected as the features. The random forest model is used to realize epilepsy detection based on the improved sample entropy and phase synchronization index. The block diagram of epilepsy detection is shown in Figure 1.

### 2.2. VMD

**Figure 2:**
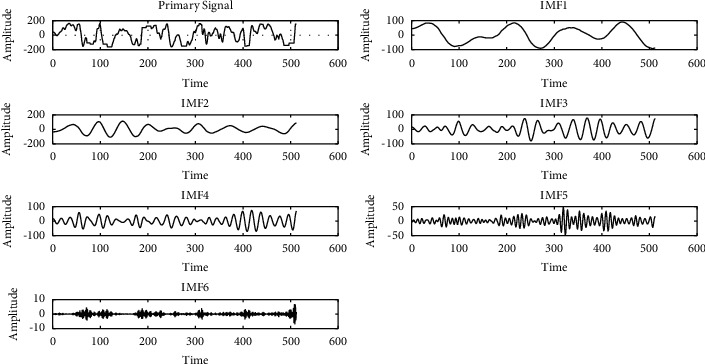
Example of VMD (the number of IMFs is 6).

VMD is a completely nonrecursive signal decomposition method, which mainly decomposes the signal into several narrow band components around different center frequencies. The center frequency is constantly changing. By finding the optimal solution of the constrained variational model, the variational modal components are obtained. The adaptive segmentation of each component in the frequency domain is completed. More details are in [12]. EEG signal can be decomposed into multiple components living in different frequency bands by VMD in the paper. An example of VMD is shown in Figure 2.

### 2.3. Improved Sample Entropy

The paper proposes a sample entropy improving method which can represent signal complexity in noisy environment truly. The processing contains nonuniform processing and adjustment.

#### 2.3.1. Principle of Sample Entropy

Sample entropy is an important index to describe the complexity of signal [13]; the steps are as follows.

**Step 1 .**

The sequence *x*(*n*)(*n*=1,2,…, *N*) is composed of a group of P-dimensional vectors denoted as *X*(*l*). It is expressed as

(1) $$\begin{array}{c} X\left(l\right)=\left[x\left(l\right),x\left(l+1\right),\ldots ,x\left(l+p-1\right)\right],\\ l=1∼N-P+1, \end{array}$$

**Step 2 .**

Define the distance *D*[*X*(*l*), *X*(*s*)] between vectors *X*(*l*) and *X*(*s*) as the largest difference between the corresponding elements of the two vectors. *D*[*X*(*l*), *X*(*s*)] is expressed as follows:

(2) $$\begin{array}{c} D\left[X\left(l\right),X\left(s\right)\right]=\underset{k=0∼P-1}{\text{Max}}\left[\left|x\left(l+k\right)-x\left(s+k\right)\right|\right]. \end{array}$$

**Step 3 .**

Given threshold *R*, count the number of *D*[*X*(*l*), *X*(*s*)] less than *R* and the proportion of this number to the total *N*−*P*, which is given by

(3) $$\begin{array}{c} B_{l}^{p}\left(r\right)=\frac{M}{N-p}, \end{array}$$

where *M* is the number that satisfies *D*[*X*(*l*), *X*(*s*)] < *R*, *l* = 1∼*N* + *P* + 1, l ≠ *s*.

**Step 4 .**

Calculate the average value of *B*_*l*_^*p*^(*r*) as follows:

(4) $$\begin{array}{c} B^{p}\left(r\right)=\frac{1}{N-p}\overset{N-p}{\underset{i=1}{\sum }}B_{l}^{p}\left(r\right). \end{array}$$

**Step 5 .**

The sequence is composed of *P* + 1 vector in order, and step 1 to step 4 are repeated to get the *P* + 1 vector denoted as *B*^*p*+1^(*r*).

**Step 6 .**

The sample entropy of *x*(*n*) is denoted as

(5) $$\begin{array}{c} \mathrm{SampEn}=-\underset{N⟶\infty }{\lim}\;\ln\frac{B^{p+1}\left(r\right)}{B^{p}\left(r\right)}. \end{array}$$

#### 2.3.2. Nonuniform Processing

Compared with nonepileptic signals, the proportion of epileptic signals with high amplitude in noisy environment is larger. In order to reduce the influence of noise on the amplitude of EEG signal, the nonuniform processing method is proposed. The method can enlarge the amplitude difference between epileptic signals and nonepileptic signals. It is denoted as follows:

(6) $$\begin{array}{c} y=\left\{\begin{array}{cc} x, & \left|x\right|\leq 80,\\ \log_{10}\left|x-70\right|, & x>80,\\ \log_{10}\left|x+70\right|, & x<-80, \end{array}\right. \end{array}$$

where *x* and *y* are the signal before and after nonuniform processing, respectively.

#### 2.3.3. Sample Entropy Adjustment

The existing EEG analysis methods are to divide the signal into several periods and extract feature in each period. Sample entropy is widely used in epilepsy detection. Sample entropy is the performance of overall complexity but lacking local information [14]. Sometimes sample entropy is decided mainly by local special signal. The development of epilepsy is not sudden but a process of evolution with time. Therefore, the changing trend of local sample entropy represents the feature of the overall sample entropy to a certain extent [15]. The paper proposes a sample entropy improving method for epilepsy detection, which takes 2 seconds as an analysis period and divides 2 seconds into 8 segments. According to the changing trend of sample entropy in 8 segments, this paper adjusts the sample entropy so as to improve the epilepsy recognition ability in noisy environment. The improved sample entropy is denoted as follows:

(7) $$\begin{array}{c} \mathrm{SampE}n_{\mathrm{imp}}=\mathrm{SampEn}+\mu *\alpha -\mu *\beta , \end{array}$$

SampEn and SampEn_imp_ denote sample entropy and improved sample entropy, respectively. *a* is the nonepileptic modulation coefficient of sample entropy. When the sample entropy in each segment increases 20% for 3 consecutive times, *a* is set to 1; otherwise it is set to 0. *ß* is the epileptic modulation coefficient of sample entropy. When the sample entropy in each segment decreases 20% for 3 consecutive times, *ß* is set to 1; otherwise it is set to 0. *µ* is adjustment factor which is determined by the difference of sample entropy between epileptic signals and nonepileptic signals in specific channel.

### 2.4. Phase Synchronization

**Figure 3:**
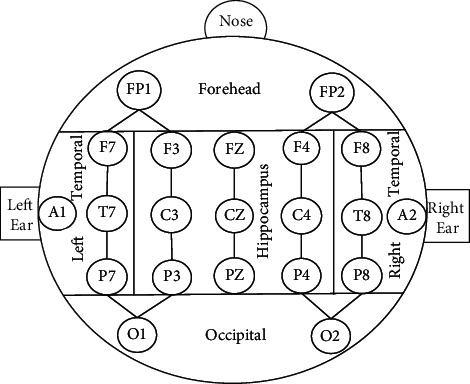
Brain region division.

It is important to select signals based on features from different regions. The original signal transmits to the scalp through complex paths. It will be affected by other signals. In the paper, the EEG signal is obtained from 5 regions based on position: forehead region, left temporal region, right temporal region, occipital region, and hippocampus region. The region division is shown in Figure 3. The signals in the same regions mostly come from the same source, so they usually have strong similarity [16].

Phase synchronization means that there is a certain relationship between 2 phases of signals. When the amplitude of the two signals remains uncorrelated, the phase of the two signals may be in a synchronous state [17]. The phase synchronization can be described as follows.

Assuming that two oscillatory systems *X* and *Y* interact with each other and the output signals of the system are *x* (*t*) and *y*(*t*), the *n*:*m* (*n*, *m* is a natural number) phase synchronization is defined where *θ*_*x*_(*t*)=*ω*_*x*_(*t*)+Φ_*x*_, *θ*_*y*_(*t*)=*ω*_*y*_(*t*)+Φ_*y*_, *ω*_*x*_/*ω*_*y*_=*m*/*n*. *θ*_*x*_(*t*) and *θ*_*y*_(*t*) are instantaneous phase of *x*(*t*) and *y*(*t*), respectively. Φ_*x*_ and Φ_*y*_ are initial phase of *x*(*t*) and *y*(*t*), respectively. *δ* is a normal number with smaller value. If *X* and *Y* have the same frequency, *ω*_*x*_/*ω*_*y*_=*m*/*n*=1, phase difference Δ*θ*_*xy*_(*t*) is denoted as Δ*θ*_*xy*_(*t*)=*θ*_*x*_(*t*) − *θ*_*y*_(*t*)=Φ_*x*_ − Φ_*y*_=ΔΦ_*xy*_.

Phase synchronization index is an important index to measure phase synchronization which is defined as follows:

(8) $$\begin{array}{c} \gamma \equiv \left|{\langle e^{j\Delta \theta_{xy}\left(t\right)}\rangle }_{t}\right|=\sqrt{{\langle \cos\left(\Delta \theta_{xy}\left(t\right)\right)\rangle }_{t}^{2}+{\langle \sin\left(\Delta \theta_{xy}\left(t\right)\right)\rangle }_{t}^{2}}, \end{array}$$

where 〈〉 means getting average.

## 3. Experiments and Results

### 3.1. Experimental Data

In this paper, the data is provided by Massachusetts Institute of Technology (MIT) [18]. The data contains 24 EEG records of 23 patients, which are collected by 10–20 international standard systems. The start time and end time of epilepsy are manually labeled by epilepsy experts. The total duration of record is 979.8 hours, including 197 records of epileptic signals lasting 3.23 hours. The data contains many types of epileptic data, which is large and representative. It is widely used for epilepsy detection.

### 3.2. Outlier Processing

**Figure 4:**
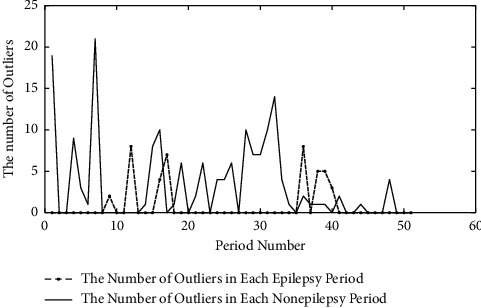
Number of outliers.

In the paper, outlier processing is used to reduce the impact of noise. 120 seconds' epileptic signals and 120 seconds' nonepileptic signals of patient 18 are randomly selected as example, respectively. 2 seconds was taken as an analysis period. Outliers in each period were identified by the Pauta criterion and replaced by median. The number of outliers is shown in Figure 4.

It can be seen from Figure 4 that the number of outliers which is got from nonepileptic signals is larger than that from epileptic signal. The main reason is that the outliers with smaller amplitude are submerged by epileptic signals with high amplitude, resulting in the reduction of outliers.

**Figure 5:**
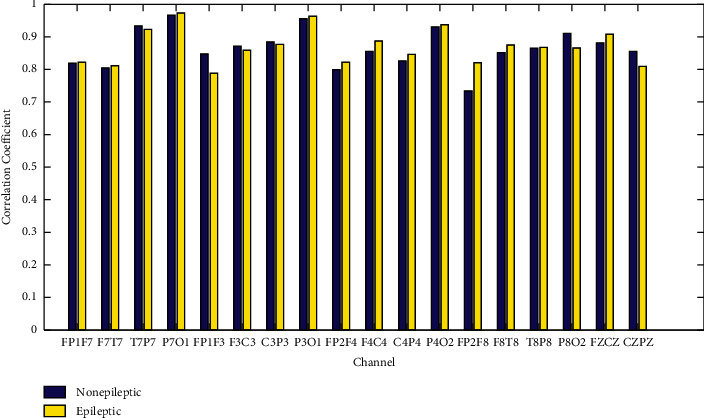
Correlation coefficients of signals between outliers processing and ICA processing.

The outliers in EEG signal usually affect the performance of epilepsy detection. Some outliers are caused by noise. Hence outlier processing is used to reduce the impact of noise. At present, ICA is widely used as one of the best denoising methods, which can remove up to 95% of the noise. Hence the signal processed by ICA is used as the reference standard. The outliers in each period were identified by the Pauta criterion. The signals are obtained by outlier processing and ICA processing, respectively. The correlation coefficient of the signals is shown in Figure 5.

It can be seen from Figure 5 that EEG signals from different channels have strong correlation with the signals after ICA denoising and outlier processing. It indicates that the outliers processing can reduce the effort of noise as ICA.

### 3.3. Improved Sample Entropy

**Figure 6:**
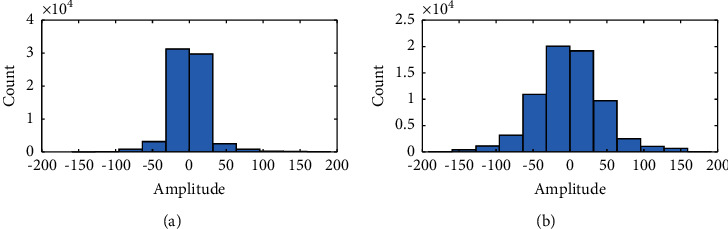
Statistics of EEG amplitude distribution: (a) Amplitude distribution of nonepileptic signals. (b) Amplitude distribution of epileptic signals.

Compared with nonepileptic signals, the amplitude of epileptic signal is relatively higher. In order to enlarge the difference between epileptic signal and nonepileptic signal, the paper adopts nonuniform processing method to process EEG signal. The paper takes signal from channel FZCZ of patient 18 as an example. The amplitude distribution of epileptic signals and nonepileptic signals is given in Figure 6. It can be seen from Figure 6 that, compared with epileptic signals, the number of nonepileptic signals with high amplitude is relatively smaller.

By enlarging the amplitude difference between epileptic signals and nonepileptic signals, it can further enlarge the difference between them, so as to complete high performance epilepsy detection.

**Figure 7:**
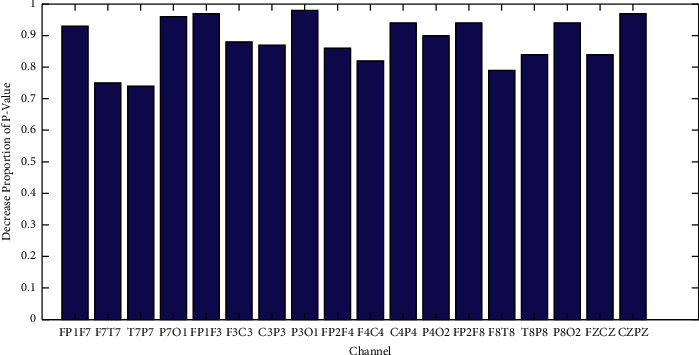
The decrease proportion of *P*-value after nonuniform processing.

The analysis of variance (ANOVA) is used to analyze the significance difference between epileptic signals and nonepileptic signals. The *P*-value decrease proportion is shown in Figure 7. The smaller the *P*-value, the stronger the ability of distinguishing epilepsy from nonepilepsy. It can be seen from Figure 7 that the significant difference decreases obviously after nonuniform processing.

The adjustment factor is decided by average sample entropy of epileptic signals and nonepileptic signals. The average sample entropy is shown in Table 1.

It can be seen from Table 1 that the relationship between sample entropy of epileptic signals and nonepileptic signals in the same channels is uncertain. The main reason is that different channels locate in different positions of brain, so they are affected by different noise and signals in other channels.

Sometimes sample entropy cannot truly represent the complexity of EEG signal because of local special signals. In order to get the complexity of local signal, the 2-second period is divided into 8 segments (represented as No 1, No 2, etc.). The sample entropy of signals in each segment is calculated respectively. The changing trend of sample entropy between 8 segments is integrated into the whole sample entropy. The partial sample entropy needs to be adjusted as given in Table 2. The sample entropy meeting the ascending adjustment requirements accounts for 4.21% of the total. The sample entropy meeting the descending adjustment standard accounts for 2.94% of the total. The sample entropy meeting the descending adjustment standard and ascending adjustment standard accounts for 0.04% of the total. The sample entropy will be adjusted based on adjustment factors when meeting adjustment standard. The adjustment factor is obtained according to average sample entropy of specific channel in Table 1. Take channel CZPZ as an example; the average sample entropy of epileptic signals is 0.66, and the average sample entropy of nonepileptic signals is 0.71. Therefore, the adjustment factor of channel CZPZ is the difference of epileptic signals and nonepileptic signals (0.05 in this case).

The ANOVA is used to analyze the significance difference of the improved sample entropy between epileptic signals and nonepileptic signals. The *P*-value is obtained by this method. The significance difference of sample entropy before and after adjustment was calculated. The results are shown in Table 3. It can be seen from Table 3 that the significance of epileptic signals and nonepileptic signals after adjustment significantly increases. In particular, the *P*-value of signals in channel F7T7 is adjusted from 0.045 to 0.023 by sample entropy adjustment. Therefore, the improved sample entropy improves the detection ability of epileptic signals and nonepileptic signals.

**Figure 8:**
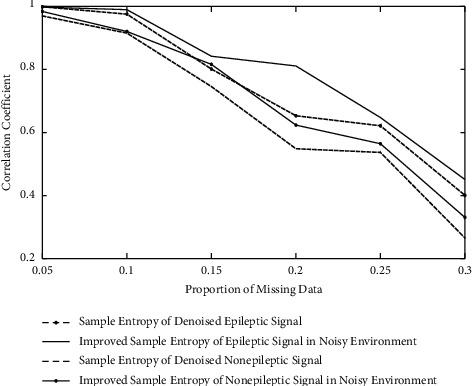
The robustness analysis of improved sample entropy.

The data acquisition environment is nonideal. Hence, data missing is inevitable in the processing of acquisition. The robustness of the improved sample entropy is analyzed in the paper. In the paper, the data which have no data missing are chosen as reference standard. The incomplete data is generated by randomly removing data in the proportion of 0.05, 0.1, 0.15, 0.2, 0.25, and 0.3. The paper calculates the correlation coefficient between incomplete data and complete data. The results are shown in Figure 8. It can be seen from Figure 8 that the correlation coefficient of the improved sample entropy in noisy environment is larger than that of the traditional sample entropy based on the denoised signals in the same proportion of data missing. This phenomenon shows that the improved sample entropy has stronger robustness than traditional sample entropy.

### 3.4. Phase Synchronization Analysis in the Same Regions

**Figure 9:**
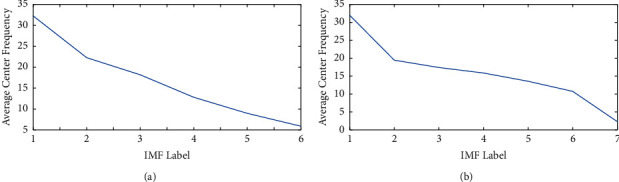
Average center frequency of every IMF: (a) The number of IMFs is 6. (b) The number of IMFs is 7.

The interaction between signals at the same frequency bands is active, which contains abundant physiological information. In order to get many IMFs at different frequency bands, the EEG signals are decomposed by VMD in the paper. It is of great significance to select the appropriate number of IMFs (denoted as K). If the K value is too small, it will produce insufficient decomposition, resulting in the neglect of meaningful information. If the K value is too large, the center frequency of different IMFs may be close to each other, resulting in mode aliasing. Hence, the center frequency observation method is adopted to get K value in [19]. The center frequency of the same IMF in different times is different. Therefore, the average center frequency is adopted by taking the average value of the center frequency of the same IMF. The paper analyzes the relationship between the number of IMFs and the average center frequency of each IMF. The results are shown in Figure 9.

It can be seen from Figure 9 that when the number of IMFs is 6, the average center frequency of each IMF has an obvious difference. However, when the number of IMFs is 7, the center frequency difference between IMFs is smaller than the number of IMFs which is 6. The phenomenon is caused by excessive decomposition of VMD. It can be concluded that 6 is the best number of IMFs in the paper.

The signals from 2 channels in the same region are decomposed to get IMFs by VMD. The average phase synchronization index of corresponding 2 IMFs in the same region is analyzed. The results are shown in Table 4.

It can be seen from Table 4 that there are some differences in the average phase synchronization index of IMFs in different regions. The farther away from the hippocampus, the lower the phase synchronization. On the whole, the phase synchronization of epileptic signals is higher than that of nonepileptic signals. The main reason is nonepileptic signals containing more random features than epileptic signals. When epileptic signals occur, the proportion of epileptic information in EEG signal becomes larger, so the degree of synchronization is higher. At the same time, with the increase of IMF's frequency, the phase synchronization index gradually decreases. The phase synchronization information carried by IMF in high frequency is significantly less than that carried by IMF in low frequency.

In the paper, the EEG of patient 18 was randomly selected for analysis with 2 seconds as analysis period. The signal is decomposed into 6 IMFs by VMD (*K* = 6). In the same region, the significance difference of phase synchronization index of corresponding 2 IMFs is analyzed. The *P*-value obtained by ANOVA is shown in Table 5.

It can be seen from Table 5 that when the phase synchronization index is used as the feature of epilepsy and nonepilepsy in noisy environment, the significance is more obvious than after signal denoising. The main reason is that when denoising method is used to remove noise, only considering amplitude and frequency but ignoring phase results in partial phase information loss. Thus, the phase synchronization index is affected. There is a significant difference in the detection ability of different IMFs of epilepsy and nonepilepsy in different region. The significance of the IMFs in the hippocampus region is obvious on the whole. This region is closest to the source of epileptic seizures. Theoretically, the epileptic information obtained is the most timely. Therefore, the phase coupling features of the hippocampus region are the best choice for phase synchronization analysis.

### 3.5. Phase Synchronization Analysis in the Different Regions

In the paper, 5 channels from 5 different regions were randomly selected for analysis. Channel FP1F3 in the forehead region, channel F7T7 in the left temporal region, F8T8 channel in the right temporal region, channel P7O1 in the occipital region, and channel FZCZ in the hippocampal region are selected as a representative channel. The data from each channel is decomposed into 6 IMFs by VMD. The ANOVA method is used for significance analysis between epileptic signals and nonepileptic signals. The results are shown in Table 6. It can be seen from Table 6 that the most of significance of epileptic signals and nonepileptic signals is obvious in different regions, but there is at least one pair of IMFs whose significance is not obvious. Therefore, the significance between epileptic signals and nonepileptic signals in the different regions is more worse than in the same regions.

### 3.6. Epilepsy Detection Results

In the paper, channel FZCZ and channel CZPZ in the hippocampus region are selected as analysis channels, and 2 seconds is taken as an analysis period. The improved sample entropy is used as features. The signals from two channels are decomposed into 6 IMFs by VMD, respectively, and phase synchronization index between corresponding IMFs (excluding IMF6) is calculated, which is a total of 7 signal features. The random forest model is used to realize epilepsy detection. Random forest is an ensemble learning algorithm, which is a representative of bagging. By combining multiple weak classifiers, the final result is obtained by voting or taking the mean value. Random forest model has high performance and generalization. The grid search method is used to optimize the model parameters. The optimal number of decision trees is 900, and the number of variables randomly selected each time is 5. In order to reduce overfitting, the 10-fold cross validation is used to complete the performance verification. Many scholars make use of the same data to achieve epilepsy detection, and the performance is shown in Table 7.

It can be seen from Table 7 that the method proposed in the paper can achieve epilepsy detection in noisy environment. From the experimental results, it can be inferred that the improved sample entropy and phase synchronization index combined with VMD can perform well as features in noisy environment. The signal loss caused by denoising is avoided, and the signal integrity is guaranteed to the greatest extent. At the same time, only two channels are selected. The 10-fold cross validation can ensure the results are independent on subject. From the 10-fold cross validation results, it can be seen that the method is effective with high accuracy. At the same time, the method can detect most of epilepsy and only very few nonepileptic signals are classified as epileptic signal.

## 4. Conclusion

Epilepsy detection is realized in noisy environment which can avoid information loss generated by denoising. The improved sample entropy and phase synchronization index are selected as features in the paper. The improved sample entropy has stronger epilepsy detection ability than sample entropy through nonuniform processing and adjustment. The channels in the same region can act better than in the different region when used for phase synchronization analysis. VMD is used to adaptively decompose the signal into 6 IMFs, and the phase synchronization indexes between corresponding 2 IMFs (excluding IMF6) can distinguish epilepsy from nonepilepsy. The random forest model realizes epilepsy detection. The results show that the accuracy, sensitivity, and specificity are 91.78%, 91.27%, and 93.61%, respectively. The results verify the advantage of the paper. That is, the method can still detect epilepsy with high performance based on EEG signal contained by complex noise. Because of the lack of epilepsy information caused by filtering, some epilepsy cannot be detected. The method effectively avoids the delay of diagnosis time which is caused by the false epilepsy detection. Hence, the method has a wider application potential.

However, the method has a disadvantage. That is, channel CZPZ and channel FZCZ do not always contain enough epilepsy information used for detecting, especially for some refractory epilepsy. In some special period, the 2 channels are not optimal channels when the quality of the two channels is poor. Therefore, establishment of adaptive unfixed channel selection method can further improve the performance of epilepsy detection through the improvement of local performance. The adaptive channel selection method in noisy environment will be our research work in the future.

## Figures and Tables

**Table 1:** Average sample entropy of epileptic signals and nonepileptic signals.

Epileptic signals				Nonepileptic signals			
Channel	Average	Channel	Average	Channel	Average	Channel	Average
FP1F7	0.66	F4C4	0.59	FP1F7	0.71	F4C4	0.70
F7T7	0.78	C4P4	0.61	F7T7	0.67	C4P4	0.75
T7P7	0.86	P4O2	0.66	T7P7	0.76	P4O2	0.71
P7O1	0.83	FP2F8	0.79	P7O1	0.79	FP2F8	0.89
FP1F3	0.75	F8T8	0.86	FP1F3	0.66	F8T8	0.81
F3C3	0.60	T8P8	0.95	F3C3	0.65	T8P8	0.84
C3P3	0.65	P8O2	0.91	C3P3	0.70	P8O2	0.97
P3O1	0.73	FZCZ	0.45	P3O1	0.81	FZCZ	0.58
FP2F4	0.61	CZPZ	0.71	FP2F4	0.64	CZPZ	0.66

**Table 2:** Examples of sample entropy meeting the adjustment standard.

No. 1	No. 2	No. 3	No. 4	No. 5	No. 6	No. 7	No. 8	Whole	Continuous ascending	Continuous descending
0.24	0.21	0.12	0.25	0.35	0.52	0.53	0.33	0.36		Yes
0.14	0.18	0.25	0.41	0.17	0.44	0.16	0.03	0.21		Yes
0.14	0.33	0.46	0.56	0.62	0.25	0.35	0.67	0.35		Yes
0.57	0.43	0.41	0.13	0.37	0.53	0.99	0.35	0.37		Yes
0.07	0.11	0.66	1.06	0.84	0.59	0.38	0.38	0.25		Yes
0.07	0.65	0.38	0.22	0.08	0.34	0.26	0.31	0.32	Yes	
1.15	0.42	0.31	0.07	0.14	0.10	0.24	0.34	0.29	Yes	
0.34	0.54	0.84	0.53	0.33	0.07	0.35	0.66	0.37	Yes	
0.73	0.21	0.40	0.83	0.76	0.54	0.42	0.20	0.38	Yes	
1.03	0.67	0.48	0.34	0.55	0.90	1.41	1.50	0.77	Yes	Yes

**Table 3:** Comparison of significance difference before and after adjustment (*P*-value).

Channel	Before adjustment	After adjustment	Channel	Before adjustment	After adjustment	Channel	Before adjustment	After adjustment
FP1F7	6.24*∗*10^−4^	1.05*∗*10^−4^	C3P3	2.14*∗*10^−15^	2.33*∗*10^−15^	FP2F8	5.61*∗*10^−7^	5.17*∗*10^−7^
F7T7	0.46	0.023	P3O1	1.48*∗∗*10^−4^	1.48*∗*10^−4^	F8T8	9.25*∗*10^−5^	8.16*∗*10^−5^
T7P7	8.21*∗*10^−3^	6.28*∗*10^−3^	FP2F4	9.56*∗*10^−6^	9.56*∗*10^−6^	T8P8	0.46	0.41
P7O1	0.26	0.20	F4C4	2.88*∗*10^−15^	2.51*∗*10^−15^	P8O2	6.26*∗*10^−11^	6.26*∗*10^−11^
FP1F3	7.02*∗*10^−6^	6.25*∗*10^−6^	C4P4	2.15*∗*10^−11^	1.77*∗*10^−11^	FZCZ	1.48*∗*10^−12^	1.11*∗*10^−12^
F3C3	0.012	9.33*∗*10^−3^	P4O2	8.80*∗*10^−6^	8.80*∗*10^−6^	CZPZ	7.93*∗*10^−30^	7.25*∗*10^−30^

**Table 4:** Average of phase synchronization index of corresponding 2 IMFs.

Region	Type	IMF 1	IMF 2	IMF 3	IMF 4	IMF 5	IMF 6
Forehead region (FP1F3–FP2F4)	Epileptic	0.94	0.92	0.87	0.71	0.33	0.24
	Nonepileptic	0.90	0.84	0.76	0.62	0.44	0.14

Left temporal region (F7T7–T7P7)	Epileptic	0.93	0.90	0.80	0.67	0.39	0.31
	Nonepileptic	0.87	0.77	0.64	0.52	0.32	0.17

Right temporal region (F8T8–T8P8)	Epileptic	0.95	0.90	0.78	0.61	0.32	0.3
	Nonepileptic	0.86	0.76	0.64	0.54	0.22	0.15

Occipital region (P7O1–P4O2)	Epileptic	0.93	0.91	0.80	0.43	0.48	0.51
	Nonepileptic	0.87	0.81	0.76	0.52	0.26	0.16

Hippocampus region (FZCZ–CZPZ)	Epileptic	0.97	0.95	0.91	0.77	0.23	0.26
	Nonepileptic	0.91	0.86	0.79	0.78	0.40	0.19

**Table 5:** Significance difference of signals in the same brain region (*P*-value).

Region	Type	IMF 1	IMF 2	IMF 3	IMF 4	IMF 5	IMF 6
Forehead region (FP1F3–FP2F4)	Denoisednoisy	2.65*∗*10^−4^	8.87*∗*10^−12^	8.89*∗*10^−6^	0.75	2.3*∗*10^−7^	2.82*∗*10^−8^
		4.98*∗*10^−4^	5.2*∗*10^−11^	5.0*∗*10^−6^	0.5064	3.0*∗*10^−7^	6.3*∗*10^−9^

Left temporal region (F7T7–T7P7)	Denoisednoisy	2.75*∗*10^−6^	4.62*∗*10^−9^	2.4*∗*10^−5^	6.3*∗*10^−4^	0.0016	1.36*∗*10^−6^
		9.6*∗*10^−8^	4.0*∗*10^−9^	8.63*∗*10^−5^	0.0025	2.0*∗*10^−5^	4.5*∗*10^−13^

Right temporal region (F8T8–T8P8)	Denoisednoisy	0.3533	0.0113	0.2365	0.2265	7.89*∗*10^−23^	8.95*∗*10^−53^
		0.443	0.0125	0.2564	0.1812	8.5*∗*10^−23^	2.5*∗*10^−53^

Occipital region (P7O1–P4O2)	Denoisednoisy	1.49*∗*10^−5^	7.04*∗*10^−10^	0.0193	0.0034	3.7*∗*10^−12^	7.48*∗*10^−43^
		5.0*∗*10^−5^	1.24*∗*10^−10^	0.0279	0.0254	2.27*∗*10^−11^	4.29*∗*10^−45^

Hippocampus region (FZCZ–CZPZ)	Denoisednoisy	6.42*∗*10^−4^	3.44*∗*10^−4^	1.53*∗*10^−8^	0.0011	6.85*∗*10^−14^	0.2568
		9.98*∗*10^−5^	1.80*∗*10^−7^	3.36*∗*10^−6^	0.0038	6.18*∗*10^−12^	0.2082

**Table 6:** Significance difference of signals in different brain regions (*P*-value).

Channel	IMF 1	IMF 2	IMF 3	IMF 4	IMF 5	IMF 6
F7T7–P3O1	1.62*∗*10^−6^	1.11*∗*10^−7^	0.0341	2.99*∗*10^−8^	0.7576	4.19*∗*10^−5^
F7T7–FP2F4	0.0014	3.61*∗*10^−5^	0.0564	0.9567	0.0173	0.0011
F7T7–T8P8	0.0095	6.57*∗*10^−4^	0.465	5.72*∗*10^−4^	0.1283	4.99*∗*10^−4^
F7T7–FZCZ	1.47*∗*10^−5^	3.09*∗*10^−7^	0.0423	0.2848	0.1465	1.94*∗*10^−6^
P3O1–T8P8	0.0013	1.70*∗*10^−4^	0.9580	1.11*∗*10^−14^	0.065	9.1*∗*10^−5^
P3O1–FZCZ	3.43*∗*10^−8^	7.80*∗*10^−15^	0.1801	1.91*∗*10^−14^	0.2255	7.21*∗*10^−4^
P3O1–FP2F4	7.46*∗*10^−5^	8.81*∗*10^−12^	0.0996	1.41*∗*10^−16^	0.2695	6.07*∗*10^−7^
FP2F4–T8P8	0.0364	0.0022	0.5932	2.34*∗*10^−5^	0.4861	8.94*∗*10^−6^
FP2F4–FZCZ	2.79*∗*10^−5^	3.36*∗*10^−10^	0.0184	0.8411	5.62*∗*10^−19^	0.0018
T8P8–FZCZ	0.0017	8.51*∗*10^−6^	0.8036	3.46*∗*10^−8^	0.9007	2.6*∗*10^−8^

**Table 7:** Epilepsy detection performance (100%).

	Accuracy	Sensitivity	Specificity	Number of channel	Noisy
Reference [20]	85.6	91.7	80.6	18	No
Reference [21]	91.09	87.83	94.35	18	No
Reference [22]	95.00	97.50	95.00	18	No
Reference [23]	95.71	98.65	84.15	23	No
Reference [24]	99.05	95.45	99.10	5	No
Reference [25]	99.6	100	99.8	23	No
Reference [26]	99.63	97.84	99.63	5	No
Reference [27]	99.66	99.72	99.60	8	No
Reference [28]	72.10	74.78	69.34	1	No
Reference [29]	75.21	50.96	87.37	1	No
Reference [30]	92.13	87.10	94.65	1	No
Reference [31]	92.79	93.07	94.84	1	No
Proposed method	91.78	91.27	93.61	2	Yes

## Data Availability

The data used to support the findings of this study are available from the corresponding author upon request.
